# Why not Intravenous Thrombolysis in Patients with Recurrent Stroke within 3 Months?

**DOI:** 10.14336/AD.2017.0406

**Published:** 2018-04-01

**Authors:** Chuanjie Wu, Di Wu, Jian Chen, Chuanhui Li, Xunming Ji

**Affiliations:** ^1^Department of neurology, Xuanwu Hospital Capital Medical University, Beijing, China; ^2^China-America Institute of Neuroscience, Xuanwu Hospital Capital Medical University, Beijing, China; ^3^Department of neurosurgery, Xuanwu Hospital Capital Medical University, Beijing, China

**Keywords:** intravenous thrombolysis, recurrent stroke, acute ischemic stroke

## Abstract

Acute ischemic stroke continues to be a very severe disorder that has significant impact on human health. Its treatment options are limited and alteplase remains the only American Food and Drug Administration-approved drug for patients with acute ischemic stroke. Furthermore, intravenous thrombolysis remains substantially underutilized, because it has rigorous indications and contraindications. Most patients simply do not meet these criteria and cannot receive thrombolytic treatment. Guidelines in many countries currently include a history of stroke within months as one of the exclusion criteria for intravenous thrombolysis. Although this is based on previous data, it lacks strong evidentiary support. Several recent studies suggested that intravenous thrombolysis may be beneficial for this patient population. We reviewed relevant publications of intravenous thrombolysis or repeated intravenous thrombolysis in patients with a history of stroke in the past 3 months. We found that intravenous thrombolysis in these patients is not as hazardous as previously believed. Among patients with relatively small infarctions and a good prognosis, intravenous thrombolysis may be a good treatment option. We hope that more research will be carried out on this topic to reexamine the criteria for intravenous thrombolysis to allow more patients to benefit from treatment.

The prevalence of stroke is high in developing and developed countries, and it is one of the leading causes of morbidity and mortality. Using intravenous thrombolysis to recanalize occluded vessels in eligible patients is recommended by current guidelines. In this article, we focus on the intravenous thrombolysis in patients with recurrent stroke within 3 months.

## Methods

Studies of intravenous thrombolysis or repeated intravenous thrombolysis in patients with a history of stroke in the past 3 months were identified from PubMed (January 1995 - September 2016). We identified relevant studies using the relevant text words and medical subject headings about stroke, cerebrovascular disease, cerebral vascular occlusion, repeated intravenous thrombolysis, intravenous thrombolysis, recurrent, alteplase, urokinase. In addition, we examined the reference lists and related links of retrieved articles in PubMed to detect studies potentially eligible for inclusion.

### The Problem

According to the 2004-2005 Report on National Survey of Mortality Causes in China, the mortality rate for stroke has exceeded that of cancer and cardiovascular disease. It has become the number one cause of death in China and the fourth in the United States. On average, one person has a stroke every 40 seconds and one dies from it every four minutes [1]. In 1996, the US Food and Drug Administration approved the intravenous application of recombinant tissue-type plasminogen activator (r-tPA) for patients with acute ischemic stroke. Since then, there are 20 years of real-world data that has repeatedly demonstrated its safety and efficacy [2]. Although there are great debates on whether combining IVT to mechanical thrombectomy in large vessel occlusion stroke, treating acute ischemic stroke patients with r-tPA intravenous thrombolysis within 4.5 h of onset remains standard management in many countries [3-5]. However, intravenous thrombolysis remains substantially underutilized. Even in US Joint Commission’s certified primary stroke centers, less than 6.7% of patients with acute ischemic stroke received r-tPA intravenous thrombolysis [1, 3, 4, 6].

There are many factors associated with this trend, including the clinician’s lack of understanding of the importance of intravenous thrombolysis and patients’ refusal of thrombolysis based on the risk for hemorrhage. However, the most important reason may be that intravenous thrombolysis has rigorous indications and contraindications, and most patients do not meet these criteria. Guidelines from the United States, Europe, Australasia, and China currently recommend that a history of stroke in the previous 3 months as a contraindication for intravenous thrombolysis [3, 4, 7]. In the United States, there are approximately 795,000 new patients with stroke or recurrent stroke annually, including 87% of patients with ischemic stroke [1]. According to data from the Oxfordshire Community Stroke Project (OCSP) and the Oxford Vascular Study (OXVASC), the rate of stroke recurrence within 3 months is as high as 14.5-18.3% [8]. It is estimated that every year there is approximately 125,000 patients with acute ischemic stroke who will not receive intravenous thrombolysis because of an occurrence of stroke within the past 3 months. Nowadays, although direct mechanical thrombectomy is an alternative in patient’s ineligible to intravenous thrombolysis, mechanical thrombectomy is only suitable for patients with proximal occlusions of the major intracranial arteries which is only eligible for about 7% of ischemic strokes [9].

The exclusion criteria of intravenous thrombolysis were established more than 20 years ago. The NINDS trial [10], which began in 1991, speculated that intravenous r-tPA thrombolysis for a recurrence acute ischemic stroke within 3 months may increase the risk of hemorrhage and considered it to be a contraindication. The results showed that patients treated with r-tPA were at least 30% more likely to have minimal or no disability at three months, compared with patients given placebo. The mortality rate of the two groups was similar, however the incidence of sICH within 36 hours was higher in the r-tPA treated group (6.4% vs 0.6%, P<0.001) [10]. Subsequent studies evaluating the efficacy of intravenous thrombolysis in patients with acute ischemic stroke in terms of time windows and dosage of r-tPA were designed to follow the exclusion criteria of NINDS trial, which were never questioned [11-13]. While the IST-3 study included 399 patients with a history of stroke within > 14 days, no outcome was reported for this group of patients [14]. Therefore, why is a history of stroke in the past 3 months an exclusion criteria, and why has it not been changed after more than 20 years?

A history of stroke in the previous 3 months used as an exclusion criterion due to the concern of bleeding for intravenous thrombolysis seems reasonable superficially. However, is it evidence-based? Is it reasonable to exclude all patients with a history of stroke in the past 3 months, no matter the severity and prognosis of the index stroke? Are we being too cautious when excluding such a large patient population who may benefit from this treatment?

Thus, there are two questions that need to be answered. First, whether intravenous thrombolysis increases the risk for hemorrhage in patients with a history of stroke in the past 3 months?Second, are there patients who can still benefit from intravenous thrombolysis?

### Does intravenous thrombolysis increase the risk for hemorrhage?

Surprisingly, the answer does not seem to be “yes.” r-tPA can directly activate plasminogen into plasmin, while thrombolysis occurs, simultaneously increasing the risk for hemorrhage [10, 12]. r-tPA is the only drug approved by the Food and Drug Administration for the treatment of patients with acute ischemic stroke, because of the benefits of recanalization. From a pathophysiology perspective, excess perfusion after recanalization of local vascular occlusion as well as various mechanisms and factors involved in ischemic/reperfusion cascade lead to the damage of the blood-brain barrier and dysfunction of the vascular basal lamina, which may cause ICH [15, 16]. Therefore, ICH can theoretically occur in the blood supply area of the occluded blood vessels. For example, in the PROACT II trial, all the ICH occurred in the infarct region [17]. In clinical practice, it is also observed that a small number of hemorrhage occurs outside the infarction after thrombolysis (<3%) [13, 18]. Post thrombolysis hemorrhage may also be related to cerebral amyloid angiopathy [19], leukoaraiosis [20], or microhemorrhages [21, 22], yet the exact mechanism remains unclear [15, 23]. Therefore, the exclusion criteria in previous studies and clinical practice may infer that the damage of blood-brain barrier and vascular basal lamina caused by previous infarction have not yet fully recovered, and thus, intravenous thrombolysis may increase the risk for hemorrhage in these areas. However, the point of contention is that the recovery of these physiological functions may not take up to three months [24]. In mouse experiments, the results showed that when the blood-brain barrier was damaged, nearby microglia could be quickly mobilized to repair the damage. In most cases, the integrity of the blood-brain barrier was restored in 10-30 min [25]. A histopathological study of patients with acute ischemic stroke showed that neutrophil infiltration in the lesion area is observed 24-48 h after infarction. Forty-eight hours post-infarction, neutrophils are gradually replaced by phagocytic cells. Ten days after infarction, the lesion area begins to liquefy, followed by decreased phagocytic cell proliferation. In the lesion area, astrocytes hyperplasia then occurs, and cyst formation starts approximately three weeks post-infraction. The blood-brain barrier is quickly destroyed in the early stages of ischemia, but can be gradually restored in a few days [26]. Although large vessel occlusion stroke and distal vessel occlusion stroke have different restoration stage theoretically, present studies did not distinguish this situation. Okada *et al.* [27] evaluated the computed tomography and angiography imaging of 160 patients with hemorrhagic transformation in cerebral embolism. The results showed that all hemorrhagic transformations occurred within 1 month. Similar results have been obtained in other studies [28, 29].

Heldner *et al.* [30] assessed 1217 patients with thrombolysis, including 17 (1.4%) patients with a stroke within the past 3 months. They found a higher proportion of symptomatic intracranial hemorrhage (sICH) in these patients (11.8% vs. 6%), a higher mortality rate (41.2% vs. 22.7%), and a lower proportion of good prognoses (29.4% vs. 48.9%). However, the baseline data were significantly different between the two groups. Thus, the authors did not compare the data statistically. In the study, the number of cases differed significantly between the two groups (17 vs. 1200). In the group of patients who had a stroke in the previous 3 months, it was noted that most of the patients had basilar artery occlusions (41.2% vs. 10.8%), and a higher mean time from symptom onset to thrombolysis (321 min vs. 262 min). We understand that the longer the time from stroke onset to treatment, the greater the risk for a secondary sICH may be associated with r-tPA [31]. Of 17 patients, seven received r-tPA intravenous thrombolysis, nine endovascular treatment, and one bridging therapy. Therefore, the aforementioned results cannot fully represent the prognosis of intravenous thrombolysis. At the same time, analyses of the hemorrhage area indicated that for patients with a history of strokes within 3 months, sICH did not occur in previous infarct areas after intravenous thrombolysis. The higher mortality in the group with recent stroke was influenced by four patients who died before discharge due to acute major index stroke. Finally, we have concluded that the poor prognosis of these patients was not attributable to their previous stroke occurrence within the past 3 months. In addition, we recommend that further evaluation of the benefit-risk ratio of intravenous thrombolysis for this group of patients should be conducted in future clinical studies. Alhazzaa *et al.* [32] analyzed all the 2008-2012 thrombolysis data from the Ottawa Hospital Research Institute. They have found that six patients had a stroke in previous three months prior to intravenous thrombolysis (3 case of repeated intravenous thrombolysis). The results showed that 3 patients developed petechial hemorrhage. The hemorrhage occurred within the area of subacute infarction, but all were asymptomatic. None of the patients had symptomatic intracranial hemorrhage or early neurological deterioration at 24 hours. At 3-month follow-up, 3 patients had an mRS of ≤2. Similar results were reported by Karlinski [33] and Kahles [34], they found that intravenous thrombolysis in patients with prior strokes in previous 3 months did not increase the risk of sICH.

However, the studies above were mostly retrospective and the samples were small. Selection bias may exist in these cases because researchers are more likely to publish results that are different from currently established findings. In other words, if ICH occurs after thrombolysis in patients with a history of stroke in the past 3 months, most researchers would take this for granted and choose not to publish the result. Therefore, conservatively, we have not noted sufficient evidence to supports the notion that patients with a history of stroke in the past 3 months have a higher risk of sICH after intravenous thrombolysis than those who did not have a previous history of stroke, especially within 3 months. By the way, the ENCHANTED study found that fewer symptomatic intracerebral hemorrhages with low-dose alteplase group compared with standard-dose group [35]. A prospective trial comparing low-dose alteplase with standard-dose in recurrent stroke is warranted.

### Can intravenous thrombolysis benefit patients with a stroke in the past 3 months?

Even though the proportion of favorable outcomes is lower [36, 37], and the mortality rate is higher in patients with repeat strokes receiving intravenous thrombolysis [38, 39], compared with patients with first-time strokes, the potential benefit of revascularization cannot be disputed. This may also be the case in patients with history of stroke within 3 months. Karlinski *et al.* [33] retrospectively analyzed the data in the Safe Implementation of Thrombolysis for Stroke database, which consisted of 13,007 cases of patients with thrombolysis from 12 countries in central and eastern Europe between October 2013 and July 2014. Overall, 11,221 (86%) patients had no history of stroke and 249 (2%) had a stroke in the past 3 months. The results showed that the proportion of patients with a good prognosis after intravenous thrombolysis (mRS 0-2), the likelihood of sICH, and 7-day and 3-month mortality rate did not significantly differ between patients with and without history of stroke in the past 3 months. Adjusted logistic regression showed that prior stroke in the past 3 months was not a risk factor for sICH, poor prognosis, or death. Subgroup analysis showed that the severity of stroke and the NIHSS score prior to intravenous thrombolysis also were not factors that influenced prognosis. The IST-3 study selected patients who had a stroke within 14 days as an exclusion criterion for intravenous thrombolysis, and the results showed that benefits and side effects of intravenous thrombolysis were not significantly different from those of previous studies using prior strokes within 3 months as an exclusion criterion [14].

### Repeat intravenous thrombolysis for recurrent stroke within 3 months

Whether a patient can benefit from repeat thrombolytic therapy within 3 months for a recurrent ischemic stroke needs further evaluation. In fact, repeat thrombolysis within 3 months may raise the concern for the risk of hemorrhage. Despite the short half-life of r-tPA (4-5 min), animal models studies have indicated that r-tPA can exacerbate the blood-brain barrier damage with additional neurotoxic effects [40].

Kahles *et al.* [34] analyzed 19 subjects with recurrent ischemic stroke within 3 months who received repeat intravenous thrombosis. These patients had an average age of 68±12 years. The median infarct volume after the first intravenous thrombolysis was 1.5 cm^3^ (interquartile range, 0.5-3.1) and median interval between the two-intravenous thrombolysis 30 days (interquartile range, 13-50). The results showed that the ratio of good prognoses after the first and second intravenous thrombolysis were 79% and 47%, respectively, and sICH was not observed in any patients after repeat thrombolytic treatments. One patient died after the second thrombolysis from the rupture of a previously unidentified infrarenal aortic aneurysm. This study suggests that repeat intravenous thrombolysis within 3 months appears to be feasible in patients with small infarction and shows a good prognosis. Sposato *et al.* [41] reported a patient with acute cerebral infarction due to atrial fibrillation, whose symptoms worsened due to an embolism 110 h after the initial intravenous thrombolysis. Thus, the patient received a repeated r-tPA intravenous thrombolysis. Unfortunately, the repeated thrombolysis caused hemorrhagic transformation of the infarction area. The patient eventually died of pneumonia and other complications 25 days after admission. However, it is worth noting that the second thrombolysis was performed 4 h after the symptoms worsened, and a standard dose of r-tPA was used.

**Table 1:** Key-studies with intravenous thrombolysis for recurrent ischemic stroke.

Author	Patient number	Previous IVT	Interval between 1^st^ and 2^nd^ events, median days	OTT in the 2^nd^ event, median min	sICH	Outcomes (mRS at 3 months)
Kahles et al. [30]	19	19	30	125	0	47% mRS≤2
Karlinski et al. [29]	249	not provided	<90	145	1.6% by SITS definition; 6.2% by ECASS II definition; No statistical difference compared with control group	48.7% mRS≤2; No statistical difference compared with control group
Heldner et al. [26]	17	not provided	46	321	11.80%	29.4% mRS≤2
Alhazzaa et al. [28]	6	3	ranging from 6 days to 10 weeks	not provided	0	50% mRS≤2
Yoo et al. [40]	2	not provided	6 and 90	420 and 70	0	both mRS were 0
Cappellari et al. [41]	3	3	2, 2, 11	35, 64, 148	0	all mRS were 0

The data from the majority of other cases supported repeat intravenous thrombolysis within 3 months or even earlier as a safe and feasible option [32, 42-45]. There are relatively fewer reports of repeated intravenous thrombolysis within 3 months (approximately 30 cases), and only one case with a poor prognosis is reported. Thus, based on the evidence available, it appears that repeated intravenous thrombolysis within 3 months is not as hazardous as imagined. Nonetheless, we should still be aware of the aforementioned publication bias. Key-studies are summarized in Table 1.

### Is it time to reconsider the inclusion criteria and exclusion criteria of intravenous thrombolysis?

More than 20 years of real-world application have accumulated a lot of data for us to analyze r-tPA intravenous thrombolysis and consider revision of the guidelines and recommendations for its application [46]. In 2012, a study of 1919 patients who did not fully meet the inclusion and exclusion criteria of intravenous thrombolysis was conducted. The results showed that general off-label r-tPA use did not correlate with sICH, mortality, and poor prognosis [47]. In a similar study, data from 500 patients who received intravenous thrombolysis were analyzed. Overall, 237 (47.4%) cases were not fully compliant with the current European r-tPA license. The results showed no significant differences of clinical outcomes at 24 h between off- and on-label thrombolysis [48]. A registry-based analysis of data form Poland indicated that off-label r-tPA use did not promote sICH, mortality, and poor prognosis. Subgroup analyses have shown a trend of increased mortality (OR 3.48, 95% CI: 0.96-12.7) and poor prognosis (OR 4.07, 95% CI: 0.97-17.1) in patients with strokes in the previous 3 months. However, the difference was not statistically significant (P = 0.058 and 0.055 respectively) [49]. Meretoja *et al*. [50] analyzed intravenous thrombolysis data from 1995 to 2008 in the Helsinki University Central Hospital. A total of 499 patients received intravenous thrombolysis, while not fully meeting the criteria recommended by the guidelines. Statistical analysis showed that intravenous thrombolysis performed outside the guideline did not increase the prospect of poor prognosis or sICH.

For patients who did not meet the criteria for intravenous thrombolysis, Frank et al. compare 1946 patients who received thrombolysis with 4285 patients who did not have the treatment. The results showed that the prognosis of patients who had intravenous thrombolysis was significantly better than those who did not [51]. These results suggest that intravenous thrombolysis performed outside the guideline is not as hazardous as imagined.

Should we consider changing some of these inclusion criteria and exclusion criteria? We are pleased to see that some changes are happening, and researchers are questioning some of these standards [52, 53] As part of prospective and retrospective studies, a number of researchers have demonstrated that the efficacy and prognosis of the treatment remains unaffected if coagulation and biochemical tests were not carried out prior to intravenous thrombolysis [54, 55]. The Japanese r-tPA intravenous thrombolysis guideline has set prior stroke in the previous 1 month as an exclusion criterion [56]. Furthermore, a history of past strokes has been completely removed from the contraindication of r-tPA, according to an updated FDA label of February 2015.

### Future directions

Although the current evidence is insufficient to address whether intravenous thrombolysis can be performed in patients with a prior stroke in the previous 3 months, it is evident that this one-size-fits-all criterion will exclude potentially eligible patients. The prognosis of intravenous thrombolysis in patients with prior strokes in the past 3 months may be related to the initial treatment of the infarction, the size and prognosis of the initial infarction, the duration between the two incidences, functional recovery (mRS) between the two strokes, the presence or not of any ICH after the first stroke and the dosage of alteplase administered for the second thrombolysis. In addition, multi-modality imaging examination may also provide guidance to thrombolysis [43, 57]. Future studies should focus on this group of the patients, and select those suitable for intravenous thrombolysis treatment. In addition, the appropriate dosage for intravenous thrombolysis, especially the repeat dosage, should also be explored.

### Conclusion

In summary, it is possible that due to the restriction of current guidelines, data on patients with recent strokes who received intravenous thrombolysis is still very limited; this is reflected in the registry database of daily clinical practices [13, 58]. However, the limited evidence showed that the administration of intravenous thrombolysis to patients with a prior stroke in the past 3 months is not nearly as hazardous as imagined. Patients with small infarction and favorable outcome should be considered for repeat intravenous thrombolysis as a treatment option. More studies are needed to address the inclusion and exclusion criteria of intravenous thrombolysis. Therefore, this effort should enable more patients with acute ischemic stroke to receive appropriate treatments.

## References

[1] Go AS, Mozaffarian D, Roger VL, Benjamin EJ, Berry JD, Blaha MJ, et al (2014). Heart disease and stroke statistics--2014 update: a report from the American Heart Association. Circulation, 129: e28-e292. 24352519 10.1161/01.cir.0000441139.02102.80 PMC5408159

[2] Harsany M, Tsivgoulis G, Alexandrov AV (2014). Intravenous thrombolysis in acute ischemic stroke: standard and potential future applications. Expert Rev Neurother, 14: 879-92. 24984941 10.1586/14737175.2014.934676

[3] Jauch EC, Saver JL, Adams HP Jr., Bruno A, Connors JJ, Demaerschalk BM, et al (2013). Guidelines for the early management of patients with acute ischemic stroke: a guideline for healthcare professionals from the American Heart Association/American Stroke Association. Stroke, 44: 870-947. 23370205 10.1161/STR.0b013e318284056a

[4] European Stroke Organisation Executive C, Committee ESOW (2008). Guidelines for management of ischaemic stroke and transient ischaemic attack 2008. Cerebrovasc Dis, 25: 457-507. 18477843 10.1159/000131083

[5] Powers WJ, Derdeyn CP, Biller J, Coffey CS, Hoh BL, Jauch EC, et al (2015). 2015 American Heart Association/American Stroke Association Focused Update of the 2013 Guidelines for the Early Management of Patients With Acute Ischemic Stroke Regarding Endovascular Treatment: A Guideline for Healthcare Professionals From the American Heart Association/American Stroke Association. Stroke, 46: 3020-35. 26123479 10.1161/STR.0000000000000074

[6] Eissa A, Krass I, Bajorek BV (2012). Barriers to the utilization of thrombolysis for acute ischaemic stroke. J Clin Pharm Ther, 37: 399-409. 22384796 10.1111/j.1365-2710.2011.01329.x

[7] Ad Hoc Committee representing the National Stroke F, the Stroke Society of A (2009). The implementation of intravenous tissue plasminogen activator in acute ischaemic stroke--a scientific position statement from the National Stroke Foundation and the Stroke Society of Australasia. Intern Med J, 39: 317-24. 19545242 10.1111/j.1445-5994.2009.01938.x

[8] Coull AJ, Rothwell PM (2004). Underestimation of the early risk of recurrent stroke: evidence of the need for a standard definition. Stroke, 35: 1925-9. 15192248 10.1161/01.STR.0000133129.58126.67

[9] Chia NH, Leyden JM, Newbury J, Jannes J, Kleinig TJ (2016). Determining the Number of Ischemic Strokes Potentially Eligible for Endovascular Thrombectomy: A Population-Based Study. Stroke, 47: 1377-80. 26987869 10.1161/STROKEAHA.116.013165

[10] (1995). Tissue plasminogen activator for acute ischemic stroke. The National Institute of Neurological Disorders and Stroke rt-PA Stroke Study Group. N Engl J Med, 333: 1581-7. 7477192 10.1056/NEJM199512143332401

[11] Hacke W, Kaste M, Bluhmki E, Brozman M, Davalos A, Guidetti D, et al (2008). Thrombolysis with alteplase 3 to 4.5 hours after acute ischemic stroke. N Engl J Med, 359: 1317-29. 18815396 10.1056/NEJMoa0804656

[12] Hacke W, Kaste M, Fieschi C, von Kummer R, Davalos A, Meier D, et al (1998). Randomised double-blind placebo-controlled trial of thrombolytic therapy with intravenous alteplase in acute ischaemic stroke (ECASS II). Second European-Australasian Acute Stroke Study Investigators. Lancet, 352: 1245-51. 9788453 10.1016/s0140-6736(98)08020-9

[13] Wahlgren N, Ahmed N, Davalos A, Ford GA, Grond M, Hacke W, et al (2007). Thrombolysis with alteplase for acute ischaemic stroke in the Safe Implementation of Thrombolysis in Stroke-Monitoring Study (SITS-MOST): an observational study. Lancet, 369: 275-82. 17258667 10.1016/S0140-6736(07)60149-4

[14] ISTc group, Sandercock P, Wardlaw JM, Lindley RI, Dennis M, Cohen G, et al (2012). The benefits and harms of intravenous thrombolysis with recombinant tissue plasminogen activator within 6 h of acute ischaemic stroke (the third international stroke trial [IST-3]): a randomised controlled trial. Lancet, 379: 2352-63. 22632908 10.1016/S0140-6736(12)60768-5 PMC3386495

[15] Alvarez-Sabin J, Maisterra O, Santamarina E, Kase CS (2013). Factors influencing haemorrhagic transformation in ischaemic stroke. Lancet Neurol, 12: 689-705. 23726850 10.1016/S1474-4422(13)70055-3

[16] Zhao H, Han Z, Ji X, Luo Y (2016). Epigenetic Regulation of Oxidative Stress in Ischemic Stroke. Aging Dis, 7: 295-306. 27330844 10.14336/AD.2015.1009 PMC4898926

[17] Furlan A, Higashida R, Wechsler L, Gent M, Rowley H, Kase C, et al (1999). Intra-arterial prourokinase for acute ischemic stroke. The PROACT II study: a randomized controlled trial. Prolyse in Acute Cerebral Thromboembolism. JAMA, 282: 2003-11. 10591382 10.1001/jama.282.21.2003

[18] Wahlgren N, Ahmed N, Davalos A, Hacke W, Millan M, Muir K, et al (2008). Thrombolysis with alteplase 3-4.5 h after acute ischaemic stroke (SITS-ISTR): an observational study. Lancet, 372: 1303-9. 18790527 10.1016/S0140-6736(08)61339-2

[19] McCarron MO, Nicoll JA (2004). Cerebral amyloid angiopathy and thrombolysis-related intracerebral haemorrhage. Lancet Neurol, 3: 484-92. 15261609 10.1016/S1474-4422(04)00825-7

[20] Nighoghossian N, Abbas F, Cho TH, Geraldo AF, Cottaz V, Janecek E, et al (2016). Impact of leukoaraiosis on parenchymal hemorrhage in elderly patients treated with thrombolysis. Neuroradiology, 58: 961-7. 27447872 10.1007/s00234-016-1725-7

[21] Kidwell CS, Saver JL, Villablanca JP, Duckwiler G, Fredieu A, Gough K, et al (2002). Magnetic resonance imaging detection of microbleeds before thrombolysis: an emerging application. Stroke, 33: 95-8. 11779895 10.1161/hs0102.101792

[22] Iqbal S, Hayman EG, Hong C, Stokum JA, Kurland DB, Gerzanich V, et al (2016). Inducible nitric oxide synthase (NOS-2) in subarachnoid hemorrhage: Regulatory mechanisms and therapeutic implications. Brain Circ, 2: 8-19. 27774520 10.4103/2394-8108.178541 PMC5074544

[23] Gu Y, Zhao K, Luan X, Liu Z, Cai Y, Wang Q, et al (2016). Association between Serum Magnesium Levels and Depression in Stroke Patients. Aging Dis, 7: 687-90. 28053818 10.14336/AD.2016.0402 PMC5198859

[24] Dongsheng H, Zhuo Z, Jiamin L, Hailan M, Lijuan H, Fan C, et al (2016). Proteomic Analysis of the Peri-Infarct Area after Human Umbilical Cord Mesenchymal Stem Cell Transplantation in Experimental Stroke. Aging Dis, 7: 623-34. 27699085 10.14336/AD.2016.0121 PMC5036957

[25] Lou N, Takano T, Pei Y, Xavier AL, Goldman SA, Nedergaard M (2016). Purinergic receptor P2RY12-dependent microglial closure of the injured blood-brain barrier. Proc Natl Acad Sci U S A, 113: 1074-9. 26755608 10.1073/pnas.1520398113 PMC4743790

[26] Kassner A, Merali Z (2015). Assessment of Blood-Brain Barrier Disruption in Stroke. Stroke, 46: 3310-5. 26463696 10.1161/STROKEAHA.115.008861

[27] Okada Y, Yamaguchi T, Minematsu K, Miyashita T, Sawada T, Sadoshima S, et al (1989). Hemorrhagic transformation in cerebral embolism. Stroke, 20: 598-603. 2718199 10.1161/01.str.20.5.598

[28] Hakim AM, Ryder-Cooke A, Melanson D (1983). Sequential computerized tomographic appearance of strokes. Stroke, 14: 893-7. 6658992 10.1161/01.str.14.6.893

[29] Hornig CR, Dorndorf W, Agnoli AL (1986). Hemorrhagic cerebral infarction--a prospective study. Stroke, 17: 179-85. 3515635 10.1161/01.str.17.2.179

[30] Heldner MR, Mattle HP, Jung S, Fischer U, Gralla J, Zubler C, et al (2014). Thrombolysis in patients with prior stroke within the last 3 months. Eur J Neurol, 21: 1493-9. 25041759 10.1111/ene.12519

[31] Fulton RL, Lees KR, Bluhmki E, Biegert G, Albers GW, Davis SM, et al (2015). Selection for delayed intravenous alteplase treatment based on a prognostic score. Int J Stroke, 10: 90-4. 23294942 10.1111/j.1747-4949.2012.00943.x

[32] Alhazzaa M, Sharma M, Blacquiere D, Stotts G, Hogan M, Dowlatshahi D (2013). Thrombolysis despite recent stroke: a case series. Stroke, 44: 1736-8. 23549131 10.1161/STROKEAHA.111.000818

[33] Karlinski M, Kobayashi A, Czlonkowska A, Mikulik R, Vaclavik D, Brozman M, et al (2015). Intravenous Thrombolysis for Stroke Recurring Within 3 Months From the Previous Event. Stroke, 46: 3184-9. 26451024 10.1161/STROKEAHA.115.010420

[34] Kahles T, Mono ML, Heldner MR, Baumgartner RW, Sarikaya H, Luft A, et al (2016). Repeated Intravenous Thrombolysis for Early Recurrent Stroke: Challenging the Exclusion Criterion. Stroke, 47: 2133-5. 27364530 10.1161/STROKEAHA.116.013599

[35] Anderson CS, Robinson T, Lindley RI, Arima H, Lavados PM, Lee TH, et al (2016). Low-Dose versus Standard-Dose Intravenous Alteplase in Acute Ischemic Stroke. N Engl J Med, 374: 2313-23. 27161018 10.1056/NEJMoa1515510

[36] Reid JM, Gubitz GJ, Dai D, Kydd D, Eskes G, Reidy Y, et al (2010). Predicting functional outcome after stroke by modelling baseline clinical and CT variables. Age Ageing, 39: 360-6. 20233732 10.1093/ageing/afq027

[37] Kong FY, Tao WD, Hao ZL, Liu M (2010). Predictors of one-year disability and death in Chinese hospitalized women after ischemic stroke. Cerebrovasc Dis, 29: 255-62. 20029199 10.1159/000267852

[38] Arboix A, Massons J, Garcia-Eroles L, Comes E, Balcells M, Oliveres M (2011). [Recurrent ischemic stroke. Study of 605 patients]. Med Clin (Barc), 137: 541-5. 21420134 10.1016/j.medcli.2010.10.027

[39] Carter AM, Catto AJ, Mansfield MW, Bamford JM, Grant PJ (2007). Predictive variables for mortality after acute ischemic stroke. Stroke, 38: 1873-80. 17446429 10.1161/STROKEAHA.106.474569

[40] Dong MX, Hu QC, Shen P, Pan JX, Wei YD, Liu YY, et al (2016). Recombinant Tissue Plasminogen Activator Induces Neurological Side Effects Independent on Thrombolysis in Mechanical Animal Models of Focal Cerebral Infarction: A Systematic Review and Meta-Analysis. PLoS One, 11: e0158848. 27387385 10.1371/journal.pone.0158848 PMC4936748

[41] Sposato LA, Salutto V, Beratti DE, Monti P, Riccio PM, Mazia C (2013). Adverse outcome of early recurrent ischemic stroke secondary to atrial fibrillation after repeated systemic thrombolysis. Case Rep Vasc Med, 2013: 371642. 10.1155/2013/371642 PMC3748420 23984177

[42] Brigo F, Bovi T, Tomelleri G, Bovi P, Moretto G (2012). Repeated systemic thrombolysis after early recurrent stroke: always hazardous? Can J Neurol Sci, 39: 114-6. 22384509 10.1017/s0317167100022137

[43] Nicoli F, Faivre A, Squarcioni C, Combaz X, Girard N (2011). Repeated MR-based intravenous thrombolysis in a patient with short interval stroke recurrences. J Neuroradiol, 38: 256-8. 21093916 10.1016/j.neurad.2010.09.003

[44] Yoo HS, Kim YD, Lee HS, Song D, Song TJ, Kim BM, et al (2013). Repeated thrombolytic therapy in patients with recurrent acute ischemic stroke. J Stroke, 15: 182-8. 24396812 10.5853/jos.2013.15.3.182 PMC3859005

[45] Cappellari M, Moretto G, Bovi P (2014). Repeated intravenous thrombolysis after recurrent stroke. A case series and review of the literature. J Neurol Sci, 345: 181-3. 25103750 10.1016/j.jns.2014.07.039

[46] Tsivgoulis G, Safouris A, Alexandrov AV (2015). Safety of intravenous thrombolysis for acute ischemic stroke in specific conditions. Expert Opin Drug Saf, 14: 845-64. 25845759 10.1517/14740338.2015.1032242

[47] Karlinski M, Kobayashi A, Mikulik R, Sanak D, Wahlgren N, Czlonkowska A, et al (2012). Intravenous alteplase in ischemic stroke patients not fully adhering to the current drug license in Central and Eastern Europe. Int J Stroke, 7: 615-22. 22309238 10.1111/j.1747-4949.2011.00733.x

[48] Cappellari M, Moretto G, Micheletti N, Donato F, Tomelleri G, Gulli G, et al (2014). Off-label thrombolysis versus full adherence to the current European Alteplase license: impact on early clinical outcomes after acute ischemic stroke. J Thromb Thrombolysis, 37: 549-56. 23943338 10.1007/s11239-013-0980-2

[49] Karlinski M, Kobayashi A, Litwin T, Sobolewski P, Fryze W, Romanowicz S, et al (2012). Intravenous thrombolysis for acute ischaemic stroke in patients not fully adhering to the European licence in Poland. Neurol Neurochir Pol, 46: 3-14. 22426757 10.5114/ninp.2012.27179

[50] Meretoja A, Putaala J, Tatlisumak T, Atula S, Artto V, Curtze S, et al (2010). Off-label thrombolysis is not associated with poor outcome in patients with stroke. Stroke, 41: 1450-8. 20538701 10.1161/STROKEAHA.109.576140

[51] Frank B, Grotta JC, Alexandrov AV, Bluhmki E, Lyden P, Meretoja A, et al (2013). Thrombolysis in stroke despite contraindications or warnings? Stroke, 44: 727-33. 23391774 10.1161/STROKEAHA.112.674622

[52] Marchidann A, Balucani C, Levine SR (2015). Expansion of Intravenous Tissue Plasminogen Activator Eligibility Beyond National Institute of Neurological Disorders and Stroke and European Cooperative Acute Stroke Study III Criteria. Neurol Clin, 33: 381-400. 25907912 10.1016/j.ncl.2015.01.004

[53] Henriksen EH, Ljostad U, Tveiten A, Naess H, Thomassen L, Mygland A (2013). TPA for ischemic stroke in patients >/=80 years. Acta Neurol Scand, 127: 309-15. 22988960 10.1111/ane.12008

[54] Rost NS, Masrur S, Pervez MA, Viswanathan A, Schwamm LH (2009). Unsuspected coagulopathy rarely prevents IV thrombolysis in acute ischemic stroke. Neurology, 73: 1957-62. 19940272 10.1212/WNL.0b013e3181c5b46d PMC4109188

[55] Sattin JA, Olson SE, Liu L, Raman R, Lyden PD (2006). An expedited code stroke protocol is feasible and safe. Stroke, 37: 2935-9. 17095736 10.1161/01.STR.0000249057.44420.4b

[56] Minematsu K, Toyoda K, Hirano T, Kimura K, Kondo R, Mori E, et al (2013). Guidelines for the intravenous application of recombinant tissue-type plasminogen activator (alteplase), the second edition, October 2012: a guideline from the Japan Stroke Society. J Stroke Cerebrovasc Dis, 22: 571-600. 23727456 10.1016/j.jstrokecerebrovasdis.2013.04.001

[57] Topakian R, Gruber F, Fellner FA, Haring HP, Aichner FT (2005). Thrombolysis beyond the guidelines: two treatments in one subject within 90 hours based on a modified magnetic resonance imaging brain clock concept. Stroke, 36: e162-4. 16210551 10.1161/01.STR.0000185799.22645.8a

[58] Hill MD, Buchan AM, Canadian Alteplase for Stroke Effectiveness Study I (2005). Thrombolysis for acute ischemic stroke: results of the Canadian Alteplase for Stroke Effectiveness Study. CMAJ, 172: 1307-12. 15883405 10.1503/cmaj.1041561 PMC557101

